# A cascade computer model for mocrobicide diffusivity from mucoadhesive formulations

**DOI:** 10.1186/s12859-015-0684-z

**Published:** 2015-08-19

**Authors:** Yugyung Lee, Alok Khemka, Gayathri Acharya, Namita Giri, Chi H. Lee

**Affiliations:** 1 School of Computing and Engineering, Kansas City, USA; 2 Division of Pharmaceutical Sciences, School of Pharmacy, University of Missouri-Kansas City, 2464 Charlotte Street, Kansas City, MO 64108 USA

**Keywords:** Computer model, Drug diffusivity, Microbicides, Mucoadhesive formulations

## Abstract

**Background:**

The cascade computer model (CCM) was designed as a machine-learning feature platform for prediction of drug diffusivity from the mucoadhesive formulations. Three basic models (the statistical regression model, the K nearest neighbor model and the modified version of the back propagation neural network) in CCM operate sequentially in close collaboration with each other, employing the estimated value obtained from the afore-positioned base model as an input value to the next-positioned base model in the cascade.

The effects of various parameters on the pharmacological efficacy of a female controlled drug delivery system (FcDDS) intended for prevention of women from HIV-1 infection were evaluated using an in vitro apparatus “Simulant Vaginal System” (SVS). We used computer simulations to explicitly examine the changes in drug diffusivity from FcDDS and determine the prognostic potency of each variable for in vivo prediction of formulation efficacy. The results obtained using the CCM approach were compared with those from individual multiple regression model.

**Results:**

CCM significantly lowered the percentage mean error (PME) and enhanced r^2^ values as compared with those from the multiple regression models. It was noted that CCM generated the PME value of 21.82 at 48169 epoch iterations, which is significantly improved from the PME value of 29.91 % at 118344 epochs by the back propagation network model. The results of this study indicated that the sequential ensemble of the classifiers allowed for an accurate prediction of the domain with significantly lowered variance and considerably reduces the time required for training phase.

**Conclusion:**

CCM is accurate, easy to operate, time and cost-effective, and thus, can serve as a valuable tool for prediction of drug diffusivity from mucoadhesive formulations. CCM may yield new insights into understanding how drugs are diffused from the carrier systems and exert their efficacies under various clinical conditions.

## Background

A variety of model-dependent procedures including Higuchi equation, a second order polynomial equation, Korsmeyer-Peppas model, Hixson Crowell, Baker-Lonsdale model, Weibull model, have been utilized for assessment of drug diffusivity from various formulations [1, 2]. The conventional model-dependent methods present acceptable proof of the intrinsic relationship between dependent and independent variables of drug diffusion and release data, but they generally lack accuracy [3]. It is evident from the previous reports that no single model is commonly employable to determine the diffusion rates of more than two drugs, if their dissolution profiles are similar to each other. A more advanced regression model based on a series of or a sequential approach is necessary to assess the association of the dependent and independent variables involved with drug diffusivity from mucoadhesive formulations.

Advances in computer technology associated with the machine learning process have brought up the vital improvements in strategies for prevention and treatment of various diseases [4, 5]. The computer based analysis techniques including artificial neural networks (ANN) and K-Nearest Neighbors Model (KNN) model made it possible to assess and predict the pharmaceutical parameters through the data mining methods [6–8]. ANN are considered as an advanced nonlinear regression tool to delineate the association of variables via iterative training of data obtained from a designed experiment [9–11]. The K nearest or K mean model is trained to find the K most similar samples in the training dataset and generates the output mean value (i.e., the most dominant output during classification) for K samples [12, 13].

In this study, the cascade computer model (CCM) was designed as a machine-learning feature platform for prediction of drug diffusivity from the mucoadhesive formulations. Three basic models (the statistical regression model, the KNN model and the modified version of the back propagation neural network) work sequentially in close collaboration with each other, employing the estimated value obtained from the afore-positioned base model as an input value to the next-positioned base model in the cascade. For example, the obtained output value which satisfies the condition that the predicted value is as close as the known output value of the hold-out dataset from KNN model (as a preliminary basic model) will be given as an initial input value to the neural network (as a secondary model) during the training process of the neural network. This approach is expected to generate the highest prediction accuracy within the least training time.

A female controlled drug delivery system (FcDDS) in the form of mucoadhesive gel has been developed as an intravaginal barrier device to prevent women from the onset of sexually transmitted disease (STD) including AIDS. Sodium dodecyl sulfate (SDS), which is a proven microbicidal agent against HIV-1 and HPV, was chosen as a model drug. The effects of various parameters on the pharmacological efficacy of FcDDS were evaluated using an in vitro apparatus “Simulant Vaginal System” (SVS) [14, 15]. The variables categorized as formulation variables (loading weight and SDS loading doses of FcDDS), intrinsic variables (vaginal fluid pH, vaginal fluid secretion rate, and rotation/vibration speed of physical movement) and extrinsic variables (inserting position), were evaluated for their prognostic potency in defining diffusivity of loaded drugs under various conditions [16, 17]. The changes in diffusivity of loaded drugs from FcDDS were explicitly examined through the computer simulation processes, and the prognostic potency of each variable for in vivo prediction of pharmacological efficacy was determined. The results obtained from CCM approach were compared with those from individual multiple regression models (i.e., Higuchi equation and a second order polynomial equation, KNN and ANN models).

Numerous theoretical issues in the machine learning analysis evolve around the tasks of finding relevant features among involved parameters. The ensemble predictive model (i.e., CCM) programmed in this study strives to achieve the highest accuracy possible within the least training time. A machine-learning feature model will yield new insights into understanding how loaded drug is diffused from the delivery systems and exerts their efficacies under various clinical conditions. A data analysis based on CCM allows each customer a self-controllable dosage regimen, which may lead to patient-specific protocols for prevention and treatment against various diseases including AIDS.

## Results

Drug diffusivities (D) from FCDDS under various conditions were assessed using CCM and other conventional regression models on randomly selected 48 records from the training dataset (sampling without any replacements).

### The regression model

The values of the PME for 10 different simulations generated by the regression models are shown in Table 1. Because the regression model tries to predict the output value as a linear function of the input parameters, the high PME value from the regression model indicates that the relationship between the input and output parameters was nonlinear. It also indicated that there were large differences between the predicted and the known (experimentally obtained) output values.

**Table 1:** The PME and r^2^ Values from the Conventional Regression Models

Model	Percentage mean error	r^2^
1	197 %	0.93
2	193 %	0.93
3	192 %	0.93
4	179 %	0.93
5	181 %	0.93
6	187 %	0.93
7	189 %	0.93
8	194 %	0.93
9	198 %	0.93
10	200 %	0.93

### K Nearest Neighbor (KNN) Model

Because KNN model is highly reliable, all the training dataset records rather than creating multiple sub-training datasets via random sampling of the training dataset were used to find the best K value. The PME value achieved by KNN model was about 34.76 % with the K and r^2^ values of 2 and 0.90, respectively. The prediction variance calculated by KNN was similar to the experimental variance in the domain where the datasets are large and the random distribution of the records is relatively close to each other. The results of this study supported that KNN model is moderately accurate in determining D values in given data sets.

### Back Propagation Artificial Neural Network (ANN)

The number of hidden units in ANN was determined by the β value (i.e., over-determination factor) whose range varied from 0.5 to 2.75, as shown in Table 2. As the value of β increased, the value of PME generally decreased. Ten different neural networks were generated (with different values for each link weight) using the randomly sampled training datasets. The network was allowed to train itself for 2*10^5^ epochs and the least PME reached by each network was recorded. As shown in Table 3, the least PME value obtained by this model was 29.91 % and the r^2^ value was remained at 0.93. The closest relationship between the input parameters and the D value was obtained at the β value of 1.5 (27 hidden units).

**Table 2:** The relationship between the PME values, β values and Different Number of Hidden Units generated from Back Propagation ANN

β	Number of hidden units	Percentage mean error
0.5	82	33.77 %
0.75	55	32.30 %
1.0	41	31.10 %
1.25	33	30.36 %
1.50	27	29.91 %
1.75	23	29.95 %
2.0	20	29.11 %
2.25	18	29.34 %
2.75	14	29.93 %

**Table 3:** The PME and r^2^ values from Back Propagation ANN

Model	Percentage mean error	r^2^ value
1	29.91 %	0.93
2	30.84 %	0.93
3	37.99 %	0.93
4	35.23 %	0.93
5	30.27 %	0.93
6	30.26 %	0.93
7	29.94 %	0.93
8	34.11 %	0.93
9	32.24 %	0.93
10	31.95 %	0.93

**Fig. 1:**
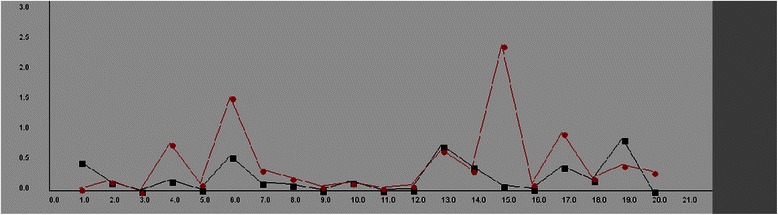
The mean errors in prediction from either Back Propagation ANN or K-Nearest Models. The solid rectangles represent the errors in prediction from back propagation ANN, whereas solid circles represent those from the K nearest neighbor model. The scale on x axis is 0.02 = 1 pixel and on y axis is 0.01 = 1 pixel

The comparative prediction error of ANN model versus KNN model for the first 20 records in the validation dataset were shown in Fig. 1. The KNN model yielded lower generalization accuracy than ANN model when the number of the records in the training dataset is larger and the relationship between the input and output parameters is not linear. Based on the results of the r^2^ and PME values, it was concluded that ANN model produces more accurate outcomes than KNN model in this study.

### The cascade computational model: a sequential ensemble of classifiers

**Fig. 2:**
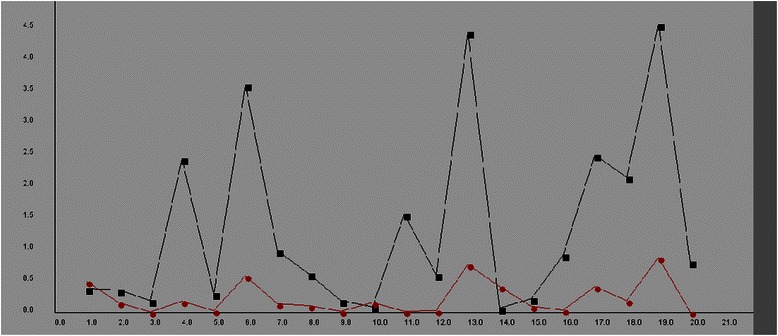
The plots of PME values from the regression model (Black) vs. the CCM model (Red)

The PME and r^2^ values obtained by CCM were shown in Table 4. The PME values were varied, ranging from 29.82 % to 37.95 %, and the r^2^ value remained as the constant value of 0.98. As shown in Table 5, both PME and r^2^ values obtained by CCM are the lowest among those by all the trained models. The β value (i.e., over-determination factor) and r^2^ value from CCM are significantly improved as compared with those generated by ANN only. The average prediction errors for the first 20 predictions by either the proposed model (red) or conventional regression approach (black) for the training dataset were shown in Fig. 2. The average prediction error and r^2^ value obtained by CCM were 14.76 % and 0.90, respectively. The high coefficient value with significantly lowered variance indicates that an accurate prediction of the domain, where the distribution profiles of the data set are similar to each other in the multidimensional space, can be achievable by CCM.

**Table 4:** The PME and r^2^ values from Sequential Ensemble of Classifiers (i.e., CCM)

Model	Percentage mean error	r^2^ Values
1	29.82 %	0.98
2	30.81 %	0.98
3	37.95 %	0.98
4	35.24 %	0.98
5	30.29 %	0.98
6	30.26 %	0.98
7	29.63 %	0.98
8	34.08 %	0.98
9	32.24 %	0.98
10	31.92 %	0.98

**Table 5:** Comparison of Prediction accuracy in PME and R^2^ values obtained by CCM and other regression models

Model	PME value	r^2^
Regression Model	179 %	0.90
K Nearest Model	34.76 %	0.90
Back Propagation Model	29.91 %	0.93
Sequential Ensemble of Classifiers	21.82 %	0.98

It was also noted that the sequential ensemble of classifiers reached the testing PME value of 21.82 at 48169 numbers of epochs as compared with the back propagation network model, which iterated for 118344 numbers of epochs for reaching the testing PME of 29.91 %. The results of this study indicated that the sequential ensemble of the classifiers considerably reduces the time required for training phase.

The summary of the D values obtained from various models was shown in Table 6, in which D value obtained from CCM was in close correlation with experimentally obtained output values. CCM produces better PME values than ANN or KNN individually, and seems to be a proper model for the accurate prediction of diffusivity of loaded drugs from FcDDS.

**Table 6:** Summary of diffusivity coefficient (D: cm^2^ hr^−1^ × 100) values obtained by various regression models

No	Higuchi	MR I	K-	Propagation	CCM	D: known output
1	4	4.5	5.3	5.56	5.26	5.00
2	3	3.5	4.3	4.55	4.35	4.30
3	3	3.5	3.4	3.59	3.40	3.50
5	4	3.5	6.3	7.69	6.59	6.50
8	3	3.5	2.6	2.58	2.50	2.50
11	3	4.5	4.0	4.08	4.00	4.00
14	2	1.5	1.0	0.20	1.20	1.40
20	3	4.0	3.1	2.43	2.51	2.50
23	4	3.0	5.4	5.90	5.55	5.50
26	3	3.0	3.2	3.45	3.50	3.50
29	3	3.0	3.4	3.42	3.50	3.50
38	3	2.5	3.0	2.64	3.20	3.00
44	2	1.5	2.1	1.66	2.25	2.00
47	3	2.5	3.5	3.12	3.00	3.00
64	2	3.0	2.6	2.57	3.15	3.00

### The validation process of the cascade computational model

**Fig. 3:**
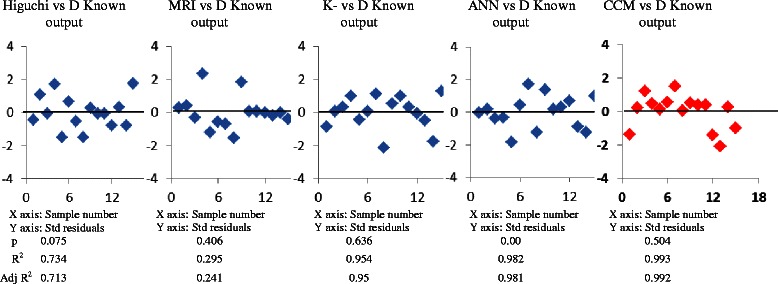
The results of the validation process on the goodness of fit and randomness of the regression residuals assessed by plotting differences between the predicted values of diffusivity coefficient (D: cm^2^ hr^−1^ × 100) from various regression models vs. experimentally obtained values

The results of the validation process on the goodness of fit and randomness of the regression residuals between the predicted values of diffusivity coefficient (D: cm^2^ hr^−1^ × 100) from various regression models vs. experimentally obtained values are shown in Fig. 3. The residuals from a fitted model are the differences between corresponding prediction of the diffusivity computed by CCM and those observed at each combined value of the involved variables. The predicted value of diffusivity obtained by CCM is much closer to the experimentally obtained value of the training dataset than those by the conventional regression methods, indicating that CCM is a suitable model for the accurate prediction of diffusivity of SDS from FcDDS. The high coefficient values of r^2^ (0.993) with even and random residual distribution by CCM suggested that an accurate prediction of the domain with significant lower variances can be achievable through application of CCM. The comparative outcome of the validation process of the proposed model attests to that the CCM produces an accurate prediction of diffusivity of loaded drugs from FcDDS and that the model can be applicable to the individual subjects for prescribing patient-specific drug regimen.

## Discussion

It is generally known that the nature of the polymer host, especially the solubility ratio (R = Cs/Co), significantly affects drug release profiles, and the magnitude of the effect is largely dependent on the drugs used in each study. The deviation from exact values for those compounds having the big solubility ratio have posed as the major constraint of the conventional model-dependent methods including an approximated Higuchi equation as compared to more advanced equations. It was found that even though the conventional model-dependent methods were effective in initial interpretation of the experimental data, to explore the advanced computational method seems to be necessary to characterize and efficiently optimize the polymer systems including a FCDDS for the controlled delivery of various microbicides.

Various computer based analysis techniques enabled us to assess and predict the pharmaceutical parameters through the machine learning process [18]. KNN model did not need parameter based simulation and was aimed to find the best feasible K value which satisfied the condition that the predicted value is as close as the known output value of the hold-out dataset [19]. ANN are networks of adaptable nodes that store experimental knowledge through the machine-based learning process from the given samples. Thus, a combination of ANN and KNN has been applicable to the establishment of a nonlinear relationship between the causal factors and the pharmacological efficacy [10].

In this study, it was hypothesized that CCM which was sequentially incorporated with KNN and ANN can be used as an advanced machine-based learning tool to predict the diffusivity of drugs from FcDDS. The classifier ensemble in a combined model improves the predictable accuracy through voting or variations thereof to reconcile models interactions. The collaborative data mining model consisted of the base models generates the predictions through a sequential input transfer action from the afore-positioned base model to the next positioned base model in the cascade. CCM first trains the statistical regression and KNN model, and selects the model with the higher prediction accuracy. During the training phase of ANN, the observed output values were also given as an input value to the network. During the prediction phase, an output value of the previously selected models (between statistical regression and k nearest model) was given to trained neural network for the final prediction.

The trial-test data set previously reported was examined with the proposed CCM. Because CCM is highly stable, all the training dataset records (instead of generating multiple sub-training dataset through the random sampling process) were used to find diffusivity of loaded microbicides in FcDDS. As ARE in percentage estimate the random errors that make the relationship between the involved variables and the outcome values (i.e., diffusivity D values), a statistical relationship will be established once the model correctly fit the data. If the residuals appear to behave randomly, it can be concluded that the model suitably fits the data, whereas if non-random structure is evident in the residuals, the model fits the data poorly. The validation of CCM assessed by comparing ARE in percentage also supported the superiority of CCM in prediction of diffusivity of loaded drugs from mucoadhesive formulations.

Because there is a unique set of variables that can be used to generate the experimental data and model simulation, the proposed computational model is somewhat constrained. This presumption is expected of the models applied to physiologically complex organs, in particular when all data were gathered under the similar conditions, which had limitation in incorporating inter-patient variance. Even though the sequential ensemble of classifiers may not be able to improve the prediction accuracy of some specific problem domains by a very large margin, CCM considerably reduces the time required for the training phase. In addition, CCM interpreted microbicidal efficacy of FcDDS from a component-oriented framework by imposing criteria determined from the EC_50_ values.

The results showed that the machine-learning feature model is a valuable tool for predicting drug diffusivity from mucoadhesive formulations. The proposed computer model can be expanded to include new variables as they become available and other factors as they become of interest. An addition of new components will be determined based on their capability to simulate and contribute the physiological and physicodynamic patterns from experimental or clinical data.

## Conclusion

An advanced computer-learning feature model (i.e., CCM) was designed for establishment of the general relationships between drug diffusivity from FcDDS and the formulation/physiological conditions at the implant site. A machine-learning feature model prospectively assessed the implication of intrinsic and extrinsic variables of FcDDS and determined the contribution capacity of each prognostic variable in predictive outcomes. A sequential ensemble of classifiers can generate continuous prediction domains with higher accuracy and spend less training time than other individual regression models. A machine-learning feature model will yield new insights into understanding how a drug diffused from the carrier systems and exert their efficacies under various clinical conditions. A data analysis based on the machine-learning CCM may allow each customer a self-controllable dosage regimen.

## Method

### Data source

The datasets were obtained through in vitro experiments on the release profiles of the model drug (i.e., SDS) from FcDDS for 6 hr using an in vitro apparatus named as “simulant vaginal system” [17]. Because SDS is intended for topical protection for women against HIV-1 during the intercourse, a period of 6 hr was selected. The data generated for this study consisted of a total of 96 datasets, out of which a half of the records (48 records) were randomly selected for the training dataset, which was used for the accurate determination of Diffusivity (D) of loaded drugs via varying trained models. The rest of the records (48 records) were used for accuracy validation.

The drug release profiles expressed as a function of tested variables were shown in Table 7, in which 15 representative results out of 96 cases were included. Because each dataset contained multiple records consisted of various combinations of input parameters, it was analyzed to extract unique patterns out of all possible outputs. In this context, a more precise measurement and new information on the detailed characteristics of such formulations can be obtained by analyzing all or a sufficient number of the individual subunits through various techniques. In addition, the generation of such multiple subunits can be properly optimized to obtain the best outputs.

**Table 7:** Variables for Diffusion Coefficient of Microbicides from Mucoadhesive formulations [17]

No	Loading dose (g/100 ml)	Gel weight (g)	pH of VFS	Flow rate (ml/hr)	Insertion Position (cm)	Q (%)
1	3	1.5	4.0	3	5	58.6
2	3	1.5	4.0	3	15	45.5
3	3	1.5	4.0	3	5	35.9
5	3	1.5	4.0	5	15	76.9
8	3	1.5	5.5	3	15	25.8
11	3	1.5	5.5	5	15	40.8
14	3	1.5	7.4	3	15	2.0
20	3	3.0	4.0	3	15	24.3
23	3	3.0	4.0	5	15	59.0
26	3	3.0	5.5	3	15	34.5
29	3	3.0	5.5	5	15	34.2
38	5	1.5	4.0	3	15	26.4
44	5	1.5	5.5	3	15	16.6
47	5	1.5	5.5	5	15	31.2
64	5	3.0	5.5	5	15	25.7

**Fig. 4:**
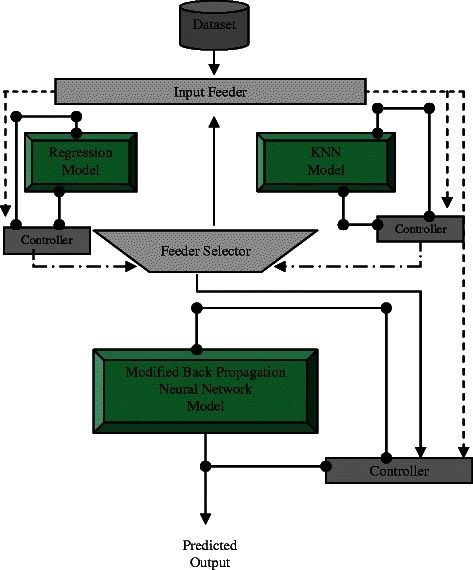
The Cascade Computer Model

CCM incorporated with a series of KNN and the back propagation ANN model was connected for the optimization process of algorithms as shown in Fig. 4. The basic diffusion formulary (i.e., Higuchi equation and a second order polynomial equation) were also performed for comparison purpose. The data were also analyzed using such individual models as the multiple regression models (MRM) [20], KNN [12, 13] and back propagation ANN model [21]. The results were recorded as an index of the drug release profiles and used for delineating the diffusivity coefficient (D) of each training data set. The equations and detailed steps taken for ANN, KNN and CCM are described in the chapter of Availability of supporting data.

### Procedure

#### Higuchi equation

The modified Higuchi equation was used to estimate the parameters of the release profiles of SDS from FcDDS. The modified Higuchi equation has frequently served as a simple regression method to obtain approximate values of parameters involved with the drug release profiles from mucoadhesive gel-or matrix-type formulations. The release profile of the drug was examined using the Higuchi equation (1);

1 $$ \mathrm{Q}=\sqrt{2*\mathrm{A}*\mathrm{C}\mathrm{s}*\mathrm{D}*\mathrm{t}} $$

in which Q is the percentage of drug released from the FcDDS at time t (in hours), A is the total concentration of drug, D is the diffusion coefficient of the drug, and C_s_ is the solubility of drug in formulations [22, 23]. The experiments were conducted under the varying conditions of the input parameters, such as dose (A), weight (w), flow rate of the physiological fluid (f rate), pH value of the physiological fluid (pH) and insertion position of the drug (iPos). The release amount (Q) of SDS was measured at predetermined interval for 6 hrs. As previously described, the D values in the equation were calculated using other available parameters whose values are already known or experimentally obtained.

#### Multiple Regression Model (MRM)

The statistical regression model is a component regression tool bundled inside the collaborative regression model. This model finds a best-fit equation out of the sample data, which satisfy the condition that the summation of the square root of the error between the predicted and actual output for the known samples is minimized (the least square method) [20]. While performing the prediction, the model uses the derived equation, feeds the unknown samples and predicts the target values based on the output equation.

Assuming that the dimensionality of input samples is N (a vector of 1 × N dimension), the output equation can be generalized as (2):

2 $$ \mathrm{y} = \mathrm{b} + {\displaystyle \sum \left({\mathrm{a}}_{\mathrm{i}}*\ {\mathrm{x}}_{\mathrm{i}}\right)} $$

where b = y intercept or bias; x = input or independent variable; a = weight of independent variable; i = ranges from 1 to N; y = predicted output.

A regression model can be developed to predict the output as a function of the input variables in a given sample data set. The notable fact about the regression model is that it needs several assumptions about the data structure. If the dataset doesn’t possess any close relationships between the independent and dependent attributes, the predicted outcome from the regression model may not be accurate. Besides, because the training of the model is memory resident (i.e. all the input sample records needs to be in the memory in a matrix form for assessment of the optimal weight), the model may not possess high scalability for a huge sample data set.

The advantages of the regression model are it’s simplicity to interpret the results and convenience as a preliminary tool. Once the model is trained, only the weight matrix needs to be in the memory to predict the output. Besides, during the training phase the model needs to iterate over the entire training data only once, which makes it a very fast learner as compared with other models in analyzing large-size datasets. Nonetheless, the operating time and space complexity of the prediction phase of the regression model need to be improved.

#### K Nearest Neighbor Model (KNN)

As previously described, the K nearest model found the K most similar samples (the most dominant output during the classification process) in the training dataset. For computing the similarity between unknown and known sample records, the Euclidean distance metric is widely used [13]. The Euclidean distance (d) between 2 points x and y may be calculated as follows (3):

3 $$ d=\sqrt{{\left(\mathrm{x}\hbox{-} \mathrm{y}\right)}^2} $$

The model training efforts are aimed at finding the optimal value for K, which mostly satisfied the condition that the root mean square (RMS) error for the testing or hold out sample is minimized. For this purpose, the K value increased from 1 to a preset maximum value and at each K value the RMS error for the holdout sample was calculated. The K value with the least RMS is considered the best possible K value. In mathematical terms, the K nearest model could be specified as (4);

4 $$ \mathrm{Y} = 1/\mathrm{k}\ *\ {\displaystyle \sum {\mathrm{y}}_{\mathrm{i}}} $$

where i ranges from 1 to K and y_i_ is the output column of the K most similar samples in the training dataset.

The K nearest model doesn’t need to make any presumptions about the structural distribution of the data, thus it’s more accurate than the statistical regression model when the distribution of the sample is random. Because it requires the estimation of the distance between every testing and known input of the K values, the model has high computational capabilities during the training and prediction processes.

#### Back propagation artificial neural network

**Fig. 5:**
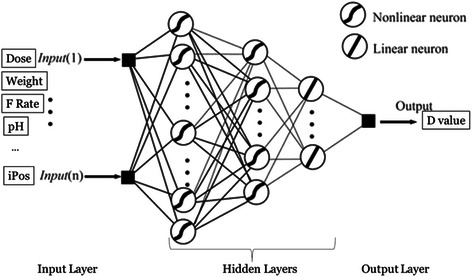
The Back Propagation Artificial Neural Network Topology

The network topology used in this study consisted of 3 layers; the input layer, the hidden layer and the output layer. The input layer consisted of 5 nodes (i.e., five input parameters) and one extra node for the bias estimation. The output layer is made of one output node, estimating the D value as shown in Fig. **5**. For the hidden layer, it consisted of one bias node and up to 27 nodes for data interaction. The number of hidden nodes in the network was determined by applying the β values from 0.5 to 2.75 in the equation (as shown in the chapter of Availability of supporting data). The β value (i.e., over determination factor), which portrays the most close relationship between the input parameters and D value, was determined and the Percentage Mean Error (PME) for the training and validation data sets, respectively, was calculated.

The activation function applied to the input layer was ‘the identity function’ that was described as follows (5),

5 $$ \mathrm{f}\left(\mathrm{x}\right) = \mathrm{x} $$

whereas the activation function applied to the hidden and output layer was ‘the sigmoid function’ that was described as follows (6),

6 $$ \mathrm{f}\left(\mathrm{x}\right) = 1\ /\ \left(1 + {\mathrm{e}}^{\hbox{-} \mathrm{x}}\right) $$

The detailed procedure for this model is described in the chapter of Availability of supporting data.

#### The Cascade Computer Model (CCM)

##### Execution of CCM

CCM consists of the three component models, namely the statistical regression model, the KNN model and the modified version of the back propagation ANN. These classifiers were combined into a unified model, whose sequential ensemble allows the model to train itself faster and more accurately, if it started with an input value close to the output value. Because the models are cascaded sequentially (i.e., the estimated value obtained from the afore-positioned base model to the later-positioned base model) rather than in parallel, no reconciliation or voting of estimations was necessary for the final prediction, eliminating the need of the output domain discretion. Thus, the training time period was shorter than those individually performed with the conventional back propagation ANN or KNN.

CCM was trained with the known parameters for maximizing its performance on determining the D value that was assigned as a dependent parameter (Table 8). The record with a higher frequency mode was selected as the D values for each dataset. For example, among three records with the same input parameter values, if 2 records had D1 value as the D value and one had D2 value as the D value, the record with the D1 was selected for the extracted dataset. Subsequently, the released amount of a model drug can be predicted by feeding the D value and other parameters in the equation.

**Table 8:** The equations in various regression models used for the assessment of diffusion coefficient

Methods	D	N
Higuchi Equation	(2*A*Cs*D*t)**1/2	7
	D = 2.32 ± 0.80	
Multivariate Regression I	D value = 2640.1023-186.17258*dose-202.39005*weight + 250.95155*flow_rate-275.11685*ph_value-48.67687*insert_pos	6
K-Nearest Neighbors	It predicts the D value as per the entered values of independent variables	
Back Propagation Model	The network was trained for each number of hidden units for a given sample and the D value with the minimal PME was calculated.	

* Statistically significant (P < 0.05)

The errors between the predicted output and the actual output (i.e., experimental values) were calculated based on the following equation (7):

7 $$ \mathrm{Error}=\frac{{\sqrt{\left(\mathrm{predicted}\_\mathrm{output}\hbox{-} \mathrm{actual}\_\mathrm{output}\right)}}^2}{\mathrm{actual}\_\mathrm{output}} $$

The square root of the difference was taken to negate the effects of the different sign between the actual and predicted outputs. After calculating the error for each test dataset, the overall error of the model (the percentage mean error (PME)) was calculated using the following equation (8):

8 $$ \mathrm{Model}\;\mathrm{Error}=\frac{{\displaystyle \sum \left(\mathrm{erro}{\mathrm{r}}_{\mathrm{i}}\right)}}{\mathrm{n}}*100 $$

Where n is the total number of records and i iterate over all the records in the dataset. The network topology was changed such that the input layer had one additional node to which the actual D value was fed during the training phase of the network. This node was fed with the predicted D value from the KNN model due to its low statistical generalization accuracy as compared with the regression model during the prediction phase of the network. The rest of the network topology remained same as the original back propagation ANN.

##### The Validation Process of the Cascade Computer model (CCM)

The rest of data (i.e., 48 studies) obtained from the previous study but not used in the computational modeling process were tested for the validation process of the proposed model. The validation procedure of CCM was executed by comparing Absolute Relative Error in percentage (ARE) obtained using the absolute value of [(Actual Output-Predicted Output)/Actual Output].

The validation process provides a proof of evidence whether the outcome is affected by a particular individual variable or an individual group of variables in the model. CCM was further modified in response to the outcomes of the validation process, if necessary, which adds the robustness of the tool in predicting the outcomes from the medical database by avoiding a spurious association within a set of variables.

## Availability of supporting data

### Model 1: Multivariate regression model

This model tries to obtain approximate values of D, diffusivity, as a function of the other independent variables, such as dose, weight, flow rate, pH and insertion position.

Steps:

1. Read all the experimental records and generate 2 matrices of those records (total of 48 records currently)
  $$ \begin{array}{l}\mathrm{Matrix}\ \mathrm{X}\ \left(48\ *\ 5\right) = \mathrm{s}\mathrm{tores}\ \mathrm{the}\ \mathrm{value}\ \mathrm{o}\mathrm{f}\ \mathrm{in}\mathrm{d}\mathrm{ependent}\ \mathrm{variables}\ \mathrm{f}\mathrm{o}\mathrm{r}\ \mathrm{all}\ \mathrm{the}\ \mathrm{r}\mathrm{ecords}.\\ {}\mathrm{Matrix}\ \mathrm{Y}\ \left(48\ *\ 1\right) = \mathrm{s}\mathrm{tores}\ \mathrm{the}\ \mathrm{value}\mathrm{s}\ \mathrm{o}\mathrm{f}\ \mathrm{all}\ \mathrm{the}\ \mathrm{d}\ \mathrm{value}\mathrm{s}\ \mathrm{f}\mathrm{o}\mathrm{r}\ \mathrm{the}\ \mathrm{r}\mathrm{espective}\ \mathrm{r}\mathrm{ecords}\ \mathrm{in}\ \mathrm{Matrix}\ \mathrm{X}.\end{array} $$
2. Iterate over all the records stored in the matrices and express the value of D as a function of the independent variables:
  $$ \mathrm{D}\_\mathrm{value}\_\mathrm{new} = \mathrm{beta}\_0 + \mathrm{dose}\ *\ \mathrm{beta}\_1 + \mathrm{weight}\ *\ \mathrm{beta}\_2 + \mathrm{f}\ \mathrm{rate}\ *\ \mathrm{beta}\_3 + \mathrm{ph}\ *\ \mathrm{beta}\_4 + \mathrm{ipos}\ *\ \mathrm{beta}\_5 $$
3. Find the values of all the beta variables, which satisfy that the summation (d_value-dvalue_new) **2 taken over all the records is minimized.
  The equation generated was:
  $$ \mathrm{D}\ \mathrm{value} = 2640.1023\ \hbox{-}\ 186.17258*\mathrm{dose}\ \hbox{-}\ 202.39005*\mathrm{weight} + 250.95155*\mathrm{flow}\_\mathrm{rate}\ \hbox{-}\ 275.11685*\mathrm{ph}\_\mathrm{value}\ \hbox{-}\ 48.67687*\mathrm{insert}\_\mathrm{p}\mathrm{o}\mathrm{s} $$

### Model 2: K-Nearest Neighbors (KNN) Model

KNN model computes the probability of the test variables belonging to a given category, which is defined based on the average value of K number of variables. Subsequently, KNN uses a category specific value for the threshold to convert the probability of the test variable belonging to a given category into a Boolean assignment. This model reads all the experimental records from the files, and waits for the user input of the independent variable values. Based on the training data readings from the files, KNN predicts the value of D in terms of the average of D values.

Steps:

1. Read all the training data from the files into the memory.
2. Accept the values of multiple independent variables from the user, at which the value of D needs to be predicted.
3. Find the multi-planar Euclidean distance between the point at which the D value needs to be predicted and the other training points at which the D value is already known.
4. Take the point at which the Euclidean distance is smallest. If there are multiple points in space with the same smallest distance, then take all of those points.
5. Find the average of D values of all the points found at step 3. This will be the final predicted D value.

It predicts the D value per entered value of independent variables.

### Model 3: Back propagation artificial network model

The number of hidden nodes in the network was determined by varying values of β in the equation (discussed in the previous case) ranging from 0.5 to 2.75. The β value most closely representing the unknown relationship between the input parameters and D value was determined.

The working of the back propagation network can be summarized as follow:

1. The signal to the input units are the input training sample fed to the network.
2. Each unit applies an activation function to its net input to calculate its output. The activation function of the input units is the identity function, i.e. y = x;
3. Each hidden layer unit calculates its net input as the weighted sum of its input from all the units in the previous layer.
4. To calculate its output the hidden unit applies the sigmoid function to it’s net input
  $$ \mathrm{y}=\frac{1}{\left(1+{\mathrm{e}}^{\hbox{-} \mathrm{x}}\right)} $$
  where x is the total input to the unit and y is the output.
5. The output unit calculates its net input as the weighted sum of the inputs from all the units in the previous hidden layer and applies the same sigmoid function to calculate the final predicted output.
  For training the network, the model iterates over all the records in the input training sample one by one (called one epoch), calculates the prediction error for each and every unit in the network till the first hidden layer, and updates the weight of the links as follows:

- For the output unit the error is calculated as:
  $$ \mathrm{Err} = \mathrm{T}\ *\ \left(1\ \hbox{--}\ \mathrm{T}\right)\ *\ \left(\mathrm{Y}\ \hbox{--}\ \mathrm{T}\right) $$
  Where T is the predicted output and Y is the actual output of the known sample.
- For the hidden layer units, the error is calculated as:
  $$ \mathrm{Err} = {\mathrm{y}}_{\mathrm{j}}*\ \left(1\ \hbox{--}\ {\mathrm{y}}_{\mathrm{j}}\right)\ *\ {\displaystyle \sum \left(\mathrm{e}\mathrm{r}{\mathrm{r}}_{\mathrm{k}}*\ {\mathrm{w}}_{\mathrm{j}\mathrm{k}}\right)} $$
  Where err_k_ is the error of the unit k in the next layer, w_jk_ is the weight of the link connecting unit j in the current layer and the unit k in the next layer and y_j_ is the output of the given hidden unit j.

Once all the errors are calculated, the weight w_jk_ of the link connecting the unit j and k is updates as:

$$ {\mathrm{W}}_{\mathrm{j}\mathrm{k}} = \mathrm{LearningRate}\ *\ \mathrm{e}\mathrm{r}{\mathrm{r}}_{\mathrm{k}}*\ {\mathrm{o}}_{\mathrm{j}} $$

Where ‘LearningRate’ is the learning rate, which determines how fast the network learns. The learning rate must be chosen wisely, because low learning rates may make the training very slow, thus very time consuming and high value of the learning rate may make the network oscillate around the optimal value of the weight.

The training of the network stops when either the error between the predicted output and actual output has reached to a predefined minimum or the model has gone through all the training records a predefined number of times called the number of epochs.The number of hidden units in the network was determined based on the equation below.

$$ {\mathrm{N}}_{\mathrm{hidden}} = \left({\mathrm{N}}_{\mathrm{sample}}/\upbeta\ \hbox{--}\ {\mathrm{N}}_{\mathrm{output}}\right)\ /\ \left({\mathrm{N}}_{\mathrm{input}} + {\mathrm{N}}_{\mathrm{output}} + 1\right) $$

where:

$$ \begin{array}{l}{\mathrm{N}}_{\mathrm{hidden}} = \mathrm{number}\ \mathrm{o}\mathrm{f}\ \mathrm{hidden}\ \mathrm{units}\\ {}{\mathrm{N}}_{\mathrm{sample}} = \mathrm{t}\mathrm{o}\mathrm{t}\mathrm{al}\ \mathrm{number}\ \mathrm{o}\mathrm{f}\ \mathrm{t}\mathrm{raining}\ \mathrm{samples}\ (154)\\ {}{\mathrm{N}}_{\mathrm{output}} = \mathrm{number}\ \mathrm{o}\mathrm{f}\ \mathrm{o}\mathrm{utput}\ \mathrm{units}\ (1)\\ {}{\mathrm{N}}_{\mathrm{input}} = \mathrm{number}\ \mathrm{o}\mathrm{f}\ \mathrm{input}\ \mathrm{units}\ (14)\\ {}\upbeta = \mathrm{o}\mathrm{verdetermination}\ \mathrm{f}\mathrm{actor}\ \left(\upbeta\ \mathrm{was}\ \mathrm{varied}\ \mathrm{f}\mathrm{rom}\ 0.5\ \mathrm{t}\mathrm{o}\ 2.75\ \mathrm{at}\ \mathrm{t}\mathrm{he}\ \mathrm{interval}\ \mathrm{o}\mathrm{f}\ 0.25\right).\\ {}\end{array} $$

The network was trained for each number of hidden units for a given sample and the training and testing PME were calculated.

### Model 4: The cascade computer model

- The cascade-computer model (CCM) can predict the drug diffusivity from FcDDS and its efficacy as a function of the external and internal physiological variables, leading to reorganization of underlying rules which govern the SDS release profiles and duration of the effective concentration. The computer based framework will provide detailed information about the involved factors, identify the efficient range of variables, and predict the microbicidal efficacy of FcDDS. A large number of release profiles of SDS (96 cases = 2^5^ × 3) obtained using SVS under various conditions along with data from animal/human studies made it possible to perform an analysis based on parameter fitting and computer networking approach. In this context, a more precise measure and new information on the detailed characteristics of such formulations can be obtained by analyzing all, or a sufficient number of the individual subunits through proposed computer-based techniques. In addition, the manufacturing of such multiple subunits can be optimized in detail to obtain the best quality product achievable.
- During the prediction phase, the collaborative model is loaded with the test samples. The input feeder of the statistical regression and k nearest model (as preliminary basic models) receives the test samples and analyzes them. The controller of that preliminary model gives its predicted output to the feeder selector, which passes it to the neural network (as a major model) controller. The network, upon being provided with the test sample input parameters as well as the predicted output (previously selected by the preliminary model) through the neural network controller (as a major model), predicts the final output for the test samples. If the required accuracy (i.e., root mean square (RMS)) hasn’t been reached, the controller again feeds the training dataset to the model and the process continues, till either the required accuracy has been reached or the maximum number of epochs allowed is elapsed.
- Pattern analysis and pattern reasoning, which are based on supervised and unsupervised learning capability from discovered patterns, will be followed to find out the correlation between variables. CCM allows integration of new and additional components into a user-friendly computing environment. Decision rules established on the ontology guides the pattern integration, which is housed in the pattern repository reserved in CCM.

## Abbreviations

D: Diffusivity
d: the Euclidean distance
A: Dose
w: weight
f rate: flow rate of the physiological fluid
iPos: insertion position of the drug
Q: and the release amount
ANN: Artificial neural networks
KNN: K-Nearest neighbors model
CCM: Cascade computer model
FcDDS: Female controlled drug delivery system
STD: Sexually transmitted disease
SDS: Sodium dodecyl sulfate
SVS: Simulant vaginal system
MRM: Multiple regression models
RMS: Root mean square
PME: Percentage mean error
ARE: Absolute relative error in percentage
